# Pharmacological Evaluation of Safoof-e-Pathar Phori- A Polyherbal Unani Formulation for Urolithiasis

**DOI:** 10.3389/fphar.2021.597990

**Published:** 2021-04-14

**Authors:** Wasim Ahmad, Mohammad Ahmed Khan, Kamran Ashraf, Ayaz Ahmad, Mohammad Daud Ali, Mohd Nazam Ansari, YT Kamal, Shadma Wahab, SM Arif Zaidi, Mohd. Mujeeb, Sayeed Ahmad

**Affiliations:** ^1^ Department of Pharmacy, Mohammed Al-Mana College for Medical Sciences, Dammam-34222, Saudi Arabia; ^2^ Bioactive Natural Product Laboratory, Department of Pharmacognosy and Phytochemistry, School of Pharmaceutical Education and Research, Jamia Hamdard, India; ^3^ Department of Pharmacology, School of Pharmaceutical Education and Research, Jamia Hamdard, India; ^4^ Faculty of Pharmacy, Universiti Teknologi MARA (UiTM), Cawangan Selangor, Kampus Puncak Alam, Selangor Darul Ehsan, Malaysia; ^5^ Department of Pharmacology and Toxicology, College of Pharmacy, Prince Sattam Bin Abdulaziz University, Alkharj, Saudi Arabia; ^6^ Department of Pharmacognosy, College of Pharmacy, King Khalid University, Abha, Saudi Arabia; ^7^ Department of Surgery, School of Unani Medical Education and Research, Jamia Hamdard, India

**Keywords:** Urolithiasis, antioxidant, ethylene glycol, ammonium chloride, safoof-e-pathar phori, long term oral toxicity

## Abstract

Safoof-e-Pathar phori (SPP) is an Unani poly-herbomineral formulation, which has for a long time been used as a medicine due to its antiurolithiatic activity, as per the Unani Pharmacopoeia. This powder formulation is prepared using six different plant/mineral constituents. In this study, we explored the antiurolithiatic and antioxidant potentials of SPP (at 700 and 1,000 mg/kg) in albino Wistar rats with urolithiasis induced by 0.75% ethylene glycol (EG) and 1% ammonium chloride (AC). Long-term oral toxicity studies were performed according to the Organization for Economic Co-operation and Development (OECD) guidelines for 90 days at an oral dose of 700 mg/kg of SPP. The EG urolithiatic toxicant group had significantly higher levels of urinary calcium, serum creatinine, blood urea, and tissue lipid peroxidation and significantly (*p* < 0.001 vs control) lower levels of urinary sodium and potassium than the normal control group. Histopathological examination revealed the presence of refractile crystals in the tubular epithelial cell and damage to proximal tubular epithelium in the toxicant group but not in the SPP treatment groups. Treatment of SPP at 700 and 1,000 mg/kg significantly (*p* < 0.001 vs toxicant) lowered urinary calcium, serum creatinine, blood urea, and lipid peroxidation in urolithiatic rats, 21 days after induction of urolithiasis compared to the toxicant group. A long-term oral toxicity study revealed the normal growth of animals without any significant change in hematological, hepatic, and renal parameters; there was no evidence of abnormal histology of the heart, kidney, liver, spleen, or stomach tissues. These results suggest the usefulness of SPP as an antiurolithiatic and an antioxidant agent, and long-term daily oral consumption of SPP was found to be safe in albino Wistar rats for up to 3 months. Thus, SPP may be safe for clinical use as an antiurolithiatic formulation.

## Introduction

Urolithiasis is the formation of a stone in the kidney, and it is the most common medical problem in humans. Kidney stones have been reported even before 4000 BC in Egyptian mummies and in remains of old North American Indians dating back to 1,500–1000 BC (Patankar et al., 2008). Kidney stones affect up to 5% of the population, with a lifetime risk of passing a kidney stone in approximately 8–10% of the population (Asplin et al., 1996). Presumably, nearly 12% of the global population is affected by kidney stones, with a higher recurrence rate in males than in females (70–80% vs 47–60%) (Soundararajan et al., 2006).

Urolithiasis is a complex process because stones can originate in any part of the urinary tract and kidney, leading to a deficiency of various vitamins and hormones in the body. The frequency of stones in men is twice as high compared to women, and the prevalence is highest among men aged between 30 and 35 years and among women aged between 35 and 55 years (Sutherland et al., 1985; Srinivas et al., 2012). Kidney stone formation or urolithiasis is a complex process that arises due to differences among promoters and inhibitors in the kidneys (Sathish et al., 2010). Factors affecting stone formation are urinary output (hence, the concentration), concentration of a particular component, urinary pH, and infection or injury within the urinary tract (Michell et al., 1989).

Stones are made up of various components. The first step in the formation of any stone is the supersaturation of urine (Chandhoke, 2002). Calcium oxalate (CaOx) is the principal constituent of most stones, accounting for more than 80% of stones (Moe, 2006), and the remaining 20% of the stone comprises struvite, cystine, uric acid, and other stones (Park and Pearle, 2007). As mentioned, calcium stone formation is caused by supersaturation of the urine with calcium salts forming a stone. Metabolic irregularities such as hypercalciuria, hypocitraturia, hyperoxaluria, hyperuricosuria, and gouty diathesis can change the composition or saturation of urine, increasing the risk of stone formation. Urolithiasis requires preventive and curative therapies because of its high recurrence rate.

Presently, no suitable drugs are available in modern medicine for the prevention and management of kidney stones. Thiazide diuretics and alkali citrate, the most effective hypocalciuric agents, are used to prevent kidney stone formation; however, no allopathic medicine is available for the dissolution or expulsion of the kidney stone (Ikshit et al., 2017). Treatment and management of renal stones relies on surgical techniques, such as extracorporeal shock wave lithotripsy, percutaneous lithotripsy, and transureteral lithotripsy (Mandavia et al., 2013; Mina et al., 2018), These surgeries are complex and expensive and do not affect the recurrence of stones. However, they cause adverse effects including tubular necrosis (Ikshit et al., 2017), hypertension, hemorrhage, and subsequent fibrosis of the kidney (Hardik et al., 2016), which cause cell injury and reappearance of the renal stone (Kaur et al., 2009).

Medicine obtained from herbal sources is a substitute and last resort drug for the prevention and treatment of kidney stones. Several medicinal plants have been used because of their curative and preventive properties against urinary calculi, for example, *Didymocarpous pedicellatus* R. Br (leaf) (Ahmad et al., 2014; Khaling et al., 2014), *Tribulus terrestris* L (fruit and leaves), *Solanum virginianum* L (fruit) (Kumar and Pandey, 2014), and Gokhsuradi churan (an ayurvedic formulation) are commonly and frequently used in traditional medicine in India (Srinivasa et al., 2013).

Traditional medicine provides several alternatives for modern invasive operational treatments in allopathy, for instance, the use of Safoof-e-Pathar phori (SPP) in the treatment of urolithiasis (Anonymous, 1986; Ahmad et al., 2016). SPP, for the treatment of urolithiasis, has been used for a long time in the Unani system of medicine and has been found to be a clinically sound medicine.

In the Unani system of medicine, powdered drugs are termed ‘Safoof’. Safoof is an important class of Unani medicinal preparations, which are obtained by powdering and mixing herbs, metals, minerals, and animal products (Dubey et al., 2008). SPP is an Unani poly-herbomineral formulation, and has been used as a medicine for a long time because of its antiurolithiatic activity, as per Unani Pharmacopoeia (Anonymous, 1986). This powder formulation is prepared using plant and mineral constituents such as *Didymocarpous pedicellatus* R. Br (Gesneriaceae) (Anonymous, 2006; Ahmad et al., 2014; Ahmad et al., 2017), *Macrotyloma biflorum* var. *biflorum* (Leguminosae) (Anonymous, 2007a), *Rheum webbianum* Royle (Polygonaceae) (Anonymous, 2007b; Ahmad W. et al., 2014), *Raphanus raphanistrum subsp. sativus* (L.) Domin (Brassicaceae), *Hordeum vulgare* Linn (Poaceae), and potassium nitrate (Ahmad et al., 2013; Ahmad W. et al., 2014; Zaidi and Ahmed, 2016).

Ahmad et al. (2016) reported extensive quality control analysis of SPP for the first time, which comprised phytochemical and physicochemical analyses, high-performance thin-layer chromatography (HPTLC), high-performance liquid chromatography (HPLC), and gas-chromatography coupled to mass spectrometry (GC-MS) fingerprinting profiles, to check the authenticity and to determine the adulterants in this traditional formulation. Quantitative estimation of marker compounds such as emodin (C_15_H_10_O_5;_ 98% purity), chrysophanic acid (C_15_H_10_O_4;_ 98% purity), and alpha-humulene (96% purity) in SPP has been performed using various analytical techniques such as HPTLC, HPLC photodiode array detector, ultra-performance liquid chromatography coupled with quadrupole time-of-flight mass spectrometry, and GC-MS (Ahmad W. et al., 2014; Ahmad et al., 2015; Ahmad et al., 2016; Ahmad et al., 2019). Additionally, a quality control analysis of individual constituents of SPP (*Didymocarpous pedicellatus* R. Br, *Macrotyloma biflorum* var. *biflorum,* and *Rheum webbianum* Royle) has been reported (Ahmad et al., 2010; Ahmad et al., 2013; Ahmad W. et al., 2014; Ahmad et al., 2017). Ahmad et al. (2020) reported a comprehensive quality assessment method consisting of HPTLC, HPLC, and GC-MS comparative fingerprinting profiles for SPP and its constituents, using extracts in petroleum ether, chloroform, methanol, and hexane to provide further chemical information. This method can be used for the identification of crude drugs present in other herbal formulations. HPTLC fingerprint profiles of petroleum ether, chloroform, and methanol extracts displayed different bands at different wavelengths for SPP and its constituents. Various solvent systems, chemical compositions, and chromatograms are reported by Ahmad et al. (2020). The HPLC finger print profile data showed the presence of 23, 14, 10, and 13 compounds was detected in methanolic extracts of SPP, *Didymocarpous pedicellatus* R. Br.*, Macrotyloma biflorum* var. *biflorum, and Rheum webbianum* Royle*,* respectively, whereas the GC-MS fingerprint profile demonstrated the presence of 22, 51, 28, and 16 metabolites in hexane extracts of SPP, *Didymocarpous pedicellatus* R. Br.*, Macrotyloma biflorum* var. *biflorum, and Rheum webbianum* Royle*,* respectively, which were identified by reviewing NIST and Wiley libraries on the basis of m/z (Ahmad et al., 2020). The formulation and composition used in this study are similar to those described by Ahmad et al. (2020).

To the best of our knowledge, no published scientific evidence is available to prove the effectiveness of SPP in urolithiasis. Hence, this study aimed to investigate phytochemical-based lithotriptic activity of SPP. In addition to investigating the therapeutic effects, obtaining comprehensive toxicological information on SPP is essential to ensure safety upon use, mainly for long-term use. Therefore, this study was conducted to evaluate the effect of SPP [same formulation as used by Ahmad et al. (2014), Ahmad W. et al. (2014), Ahmad et al. (2015), Ahmad et al. (2016), Ahmad et al. (2019), Ahmad et al. (2020)] on urolithiasis induced by ethylene glycol (EG) and ammonium chloride (AC) in albino Wistar rats and its long-term oral toxicity.

## Material and Methods

### Plant Material

Crude drugs such as *R. webbianum* Royle*, D. pedicellatus* R. Br, and *M. biflorum* var. *biflorum* were collected from the local drug market in Khari Baoli, New Delhi, and taxonomic authentication was performed by Dr. H. B. Singh, Ref. NISCAIR/RHMD/1327/129, New Delhi.

### Method of Safoof-e-Pathar Phori Preparation

SPP was prepared as per the process described in Qarabadeen Majeedi. For the 100 g unit formula for SPP preparation, finely powdered plant drugs: *Didymocarpous pedicellatus* R. Br.50 g, *Macrotyloma biflorum* var. *biflorum*12.5 g, *Rheum webbianum* Royle12.5 g, salts: *Hordeum vulgare* Linn. *12.5g, Raphanus raphanistrum subsp. sativus* (L.), Domin 6.25 g, and potassium nitrate 6.25 g were mixed properly and passed through mesh # 60 to obtain a uniform powder (Table 1). This formulation was prepared in the bioactive natural product laboratory, Jamia Hamdard, New Delhi, and a voucher specimen was submitted to the lab BNPL/SPP/02/2010. The same formulation and composition were used by Ahmad et al. (2014), Ahmad W. et al. (2014), Ahmed et al. (2015), Ahmed et al. (2019), Ahmed et al. (2020).

**TABLE 1:** Composition of Safoof-e-Pathar phori (Anonymous, 1986).

S. No	Ingredients common name	Ingredients botanical name	Family (part used)	Category	Unit formula per 100 g
	Pathar phori	*Didymocarpous pedicellatus* R.Br	Gesnericeae (leaves)	Active ingredient	50 g
	Jawakhar desi	*Hordeum vulgare* L	Poaceae (whole plant)	Active ingredient	12.5 g
	Revand chini	*Rheum webbianum Royle*	Polygonaceae (Rhizome)	Active ingredient	12.5 g
	Namak turb	*Raphanus raphanistrum subsp. sativus* (L.) domin	Brassicaceae (Whole plant)	Active ingredient	6.25 g
	Kulthi	*Macrotyloma biflorum* var. *biflorum*	Leguminosae (Seed)	Active ingredient	12.5 g
	Shora qalmi	Potassium Nitrate		Active ingredient	6.25 g
				**Total**	**100**

### Chemicals and Apparatus

Biochemical kits for the estimation of calcium, sodium, and potassium were purchased from Span Diagnostics Ltd, Surat, India. All the chemicals were purchased from Sigma Chemicals Co., St Louis, United States, and Hi-media, Mumbai. Apparatus including metabolic cages (INCO, Ambala, India), refrigerated research centrifuge (Remi centrifuge instrument, Mumbai), and UV-spectrometer (model UV 1800; Shimadzu, Japan) were used in the study.

### Experimental Animals

Male albino Wistar rats (body weight range 180–220 g) were used for the pharmacological (antiurolithiatic and antioxidant activities) studies, and healthy young adult male and female albino Wistar rats (weighing 125–160 g) were used for the long-term oral toxicity studies. Females were nulliparous and non-pregnant. Animals were procured from the central animal house facility of Jamia Hamdard. The study protocol was approved by the Institutional Animal Ethics Committee (Registration Number 173/CPCSEA and Approval Number 691) following guidelines of the Committee for the Purpose of Control and Supervision of Experiments on Animals (CPCSEA), Government of India. All animals were fed a standard laboratory diet and maintained under standard laboratory conditions (temperature, 22–24°C; relative humidity, 60–70%), standard light and dark cycle, and water *ad libitum*. All the healthy albino Wistar rats were allowed to acclimatize for 1 week prior to the experiment.

### Ethylene Glycol- and Ammonium chloride-Induced Urolithiasis

Animals were divided into the following five groups (six animals in each group): control, toxicant, Safoof-e-Pathar phori lower dose (SPPL) treatment group, Safoof-e-Pathar phori higher dose (SPPH) treatment group, and neeri syrup standard group (Table 1). To induce CaOx crystal formation, animals were exposed to 0.75% EG with 1.0% AC in their drinking water for 21 days (Bashir and Gilani, 2009). The experimental groups are summarized as follows:

Group I: Normal rats; received vehicle along with simple drinking water for 21 days.
Group II: Toxicant; received 0.75% EG with 1.0% AC in drinking water for 5 days and 0.75% EG for the next 16 days.
Group III: Standard; received 0.75% EG with 1.0% AC in drinking water for 5 days and 0.75% EG for the next 16 days along with the standard drug neeri 2.65 ml/kg/day for all 21 days.
Group IV: SPPL; received 0.75% EG with 1.0% AC in drinking water for 5 days and 0.75% EG for the next 16 days along with SPP at 700 mg/kg/day for all 21 days.
Group 5: SPPH; received 0.75% EG with 1.0% AC in drinking water for 5 days and 0.75% EG for the next 16 days along with SPP at 1,000 mg/kg/day for all 21 days.

For the antiurolithiatic study, fresh suspensions of SPP (700 and 1,000 mg/kg; using 0.25% CMC) were administered once daily by oral gavage to the corresponding group of animals for 21 days.

### Long-Term Oral Toxicity Study

Young adult male and female albino Wistar rats were divided into the following four groups (five rats in each group):

Group I: Normal control (NC); consisting of males receiving vehicle 0.4ml/animal daily for 90days.
Group II: NC; consisting of females receiving vehicle 0.4 ml/animal daily for 90 days.
Group III: SPP (700 mg/kg/day for 90 days); consisting of males.
Group IV: SPP (700 mg/kg/day for 90 days); consisting of females.

For the long-term oral toxicity study, a freshly prepared suspension of SPP (700 mg/kg) was administered daily in the rats for 90 days. The animals were dosed at approximately the same time each day. All animals were observed daily for body weight and mortality throughout the study. Histopathological and biochemical changes in the animals were analyzed at the end of the study period following sacrificing of the animals.

### Collection and Analysis of Urine

On the 21st day, animals were kept in a urine collection cage, and urine from the first 3 h of the morning was collected for investigation of crystalluria. All the animals were kept individually in the metabolic cages, and the urine sample at 24 h was collected. The urine samples were acidified by adding a few drops of concentrated HCl and stored at 4°C until further use. Urine was also analyzed for calcium, sodium, and potassium as per the manufacturer’s protocol (Span Diagnostics Ltd, Surat, India) (Ikshit et al., 2017)

### Hematological and Serum Biochemical Analysis

Blood samples were collected 24 h after the last dose of treatment by puncturing the retro-orbital sinus under a mild anesthetic condition, and the animals were sacrificed by cervical decapitation. In the pharmacological study, hematological parameters such as hemoglobin (Hb), total red blood cells (RBCs), and white blood cells (WBCs) were analyzed in the whole blood. The serum was separated by centrifugation at 1,500 rpm for 15 min in refrigerated research centrifuge and used for the estimation of serum creatinine and blood urea nitrogen (BUN) using commercially available kits (Span Diagnostics Ltd, Surat, India) and the Star-21 plus semi-auto analyzer. In long-term oral toxicity studies, hematological parameters such as Hb, erythrocyte count, total leukocytes and biochemical parameters in the blood serum including serum glutamic oxaloacetic transaminase, serum glutamic pyruvic transaminase, alkaline phosphatase, urea, albumin, bilirubin, total protein, creatinine, uric acid, sodium, and potassium were analyzed using commercially available kits (Span Diagnostics Ltd, Surat, India) as per the manufacturer’s protocol.

### Histopathological Study of the Kidney, Heart, Liver, Stomach, and Spleen

For investigating the antiurolithiatic activity, the sacrificed animals were dissected after blood sample collection, and only the kidneys were removed and immediately washed with ice-cold saline. After washing with saline, kidneys were preserved for biochemical estimation and fixed in 10% formalin for histopathological studies. For the long-term oral toxicity study, the kidney, heart, liver, stomach, and spleen of animals were dissected. Isolated organs were fixed in 10% neutral buffered formalin, processed in a series of graded alcohol and xylene, and finally embedded in paraffin wax. Histological sections (approximately 5m thickness) were prepared by microtomy and stained with hematoxylin and eosin dye for histological examination. Histological slides were examined under a light microscope at low (10×) and high (40×) magnifications.Statistical Analysis

Results are expressed as mean ± standard deviation. A two-way analysis of variance followed by a Tukey’s test was used for statistical analysis. A *p* value of <0.05 was considered statistically significant.

## Results

### Antiurolithiatic Effect of Safoof-e-Pathar Phori on Ethylene Glycol Induced Urolithiasis

The concentrations of urinary calcium, potassium, and sodium in groups I–V are given in Table 2. In this study, 0.75% EG (v/v) with 1% AC induced urolithiasis in male rats and resulted in hypercalciuria and hyperoxaluria. The level of urinary calcium was significantly elevated compared with the Normal control (NC) (Table 2). However, rats treated with SPPL (700 mg/kg; Group IV) and SPPH (1,000 mg/kg; Group V) demonstrated significantly lower calcium levels and increased potassium and sodium excretion in urine than those in NC (*p* < 0.01). A decrease in calcium and an increase in potassium and sodium in SPPL and SPPH treated rats were similar to those in Neeri-treated rats (Group III).

**TABLE 2:** Results of ions estimations in urine of control and urolithiatic group and group treated with Neeri, SPP 700 and 1000 mg/kg oral dose.

Parameter	Group I	Group II	Group III	Group IV	Group V
	Control	Toxicant	Neeri	SPP 700 mg/kg	SPP 1000 mg/kg
Calcium (mg/dl)	4.27 ± 0.12	8.90 ± 0.06^a^	5.20 ± 0.13^b^	5.95 ± 0.11^b^	5.28 ± 0.12^b^
Potassium (mg/dl)	7.10 ± 0.11	4.40 ± 0.03^a^	7.20 ± 0.07^b^	6.80 ± 0.10^b^	7.41 ± 0.06^b^
Sodium (mg/dl)	28.2 ± 0.52	10.13 ± 0.85^a^	28.1 ± 0.41^b^	25.92 ± 0.47^b^	29.92 ± 0.62^b^
Oxalate crystals	Nil	+++	Nil ^b^	Nil ^b^	Nil ^b^
BUN (mg/dl)	19.3 ± 4.5	134.7 ± 14.86^a^	27.11 ± 6.2^b^	43.26 ± 3.20^b^	23.74 ± 2.34^b^
Creatinine (mg/dl)	0.23 ± 0.02	0.69 ± 0.04^a^	0.21 ± 0.01^b^	0.27 ± 0.01^b^	0.20 ± 0.01^b^
TBARS (nmoles/mg)	3.95 ± 0.33	8.97 ± 0.51^a^	5.14 ± 0.14^b^	5.10 ± 0.37^b^	5.54 ± 0.29^b^
GSH (nmoles/mg)	3.51 ± 0.17	0.57 ± 0.11^a^	3.20 ± 0.09^b^	2.53 ± 0.04^b^	2.82 ± 0.08^b^
RBC (×10^6^/mm^3^)	5.88 ± 0.2	6.18 ± 0.3	6.33 ± 0.16	6.23 ± 0.2	6.41 ± 0.3
WBC(×10^3^/mm^3^)	9.7 ± 0.3	10.3 ± 0.2	9.7 ± 0.22	10.1 ± 0.4	10.9 ± 0.2
Hb Content (g/dl)	10.4 ± 0.3	11.7 ± 0.4	11.1 ± 0.31	11.8 ± 0.4	11.3 ± 0.3

^**a**^ *p* < 0.001 vs. Control.

^b^ *P*<0.001 vs. Toxicant a denotes that data were compared with normal control and b denotes that data were with toxicant group <0.001.

Urolithiasis induced by EG and AC caused impairment of renal functions in untreated rats as marked by the increased levels of serum creatinine, uric acid, and urea. Renal function was evaluated by determining the serum creatinine and BUN in EG- and AC-induced urolithiasis and treated rats (Table 2). Levels of serum creatinine and BUN were significantly increased in the toxicant group when compared with the NC, indicating impairment of renal functions. However, SPPL (700 mg/kg) and SPPH (1,000 mg/kg) significantly (*p* < 0.001) decreased the levels of serum creatinine and BUN excreted by kidneys compared with the toxicant group. Moreover, treatment with SPPL (700 mg/kg) and SPPH (1,000 mg/kg) significantly lowered the elevated serum creatinine level, SPPH showed dose-dependent activity due to maximum decrease in serum creatinine and BUN levels. These results suggested that SPP treatment ameliorates the renal functions compared with urolithiatic control rats. The results of SPP were found to be similar to those of the standard drug neeri.

**FIGURE 1:**
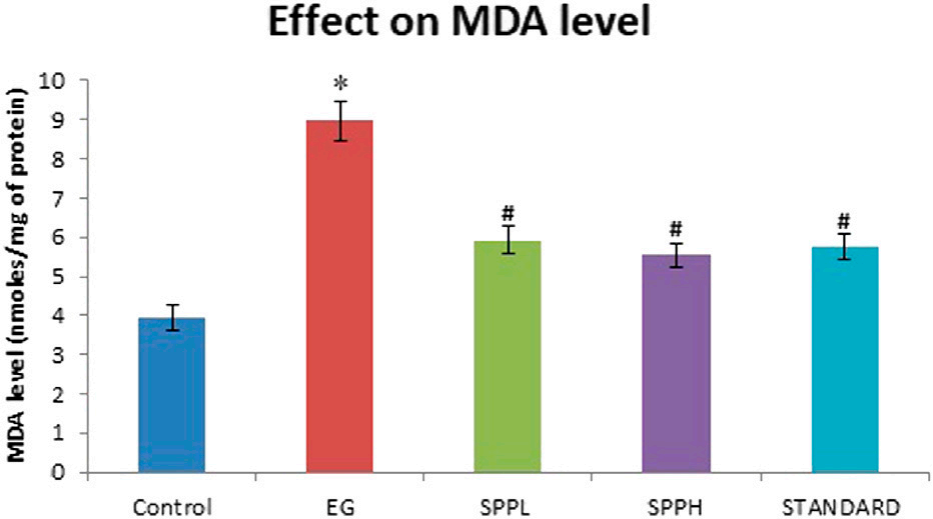
Lipid peroxidation in kidney of rats immunized with EG and AC after treatment with SPP (700 and 1,000 mg/kg b. wt.). Data are expressed as Mean ± SD of 6 rats.

**FIGURE 2:**
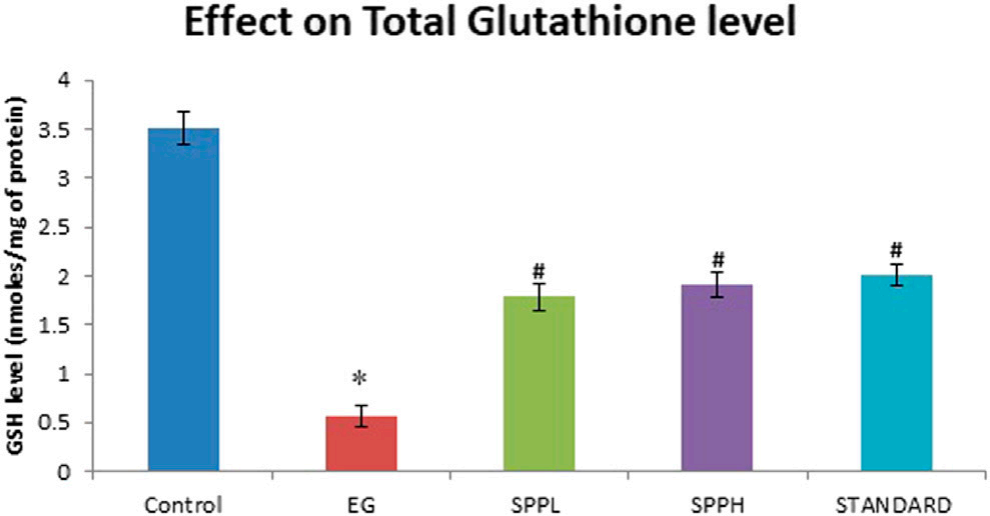
Effect of SPP treatment on GSH in the kidney of urolithiatic rats. Data are expressed as Mean ± SD of 6 rats.

The antioxidant parameters were assessed by measuring the malondialdehyde (measured as TBARS) and glutathione (GSH) level. Treatment with EG and AC significantly increased (*p* < 0.001) the TBARS level and decreased the GSH level in urolithiasis-induced rats compared with NC (Table 2). Treatment with SPPL (700 mg/kg) and SPPH (1,000 mg/kg) decreased the TBARS level significantly (Figure 1) and improved the GSH concentration (Figure 2) compared with the toxicant group.

The crystalluria study revealed the absence of crystals (oxalate crystals) in the rat group treated with standard neeri syrup (2.65 ml/kg), SPPL (700 mg/kg), and SPPH (1,000 mg/kg), which supported the preventive effect of low and high doses of SPP on the induction of urolithiasis. Hematological analysis of various groups of rats showed normal RBC, WBC, and Hb levels and absence of hemolysis in all the groups (Table 2).

**FIGURE 3:**
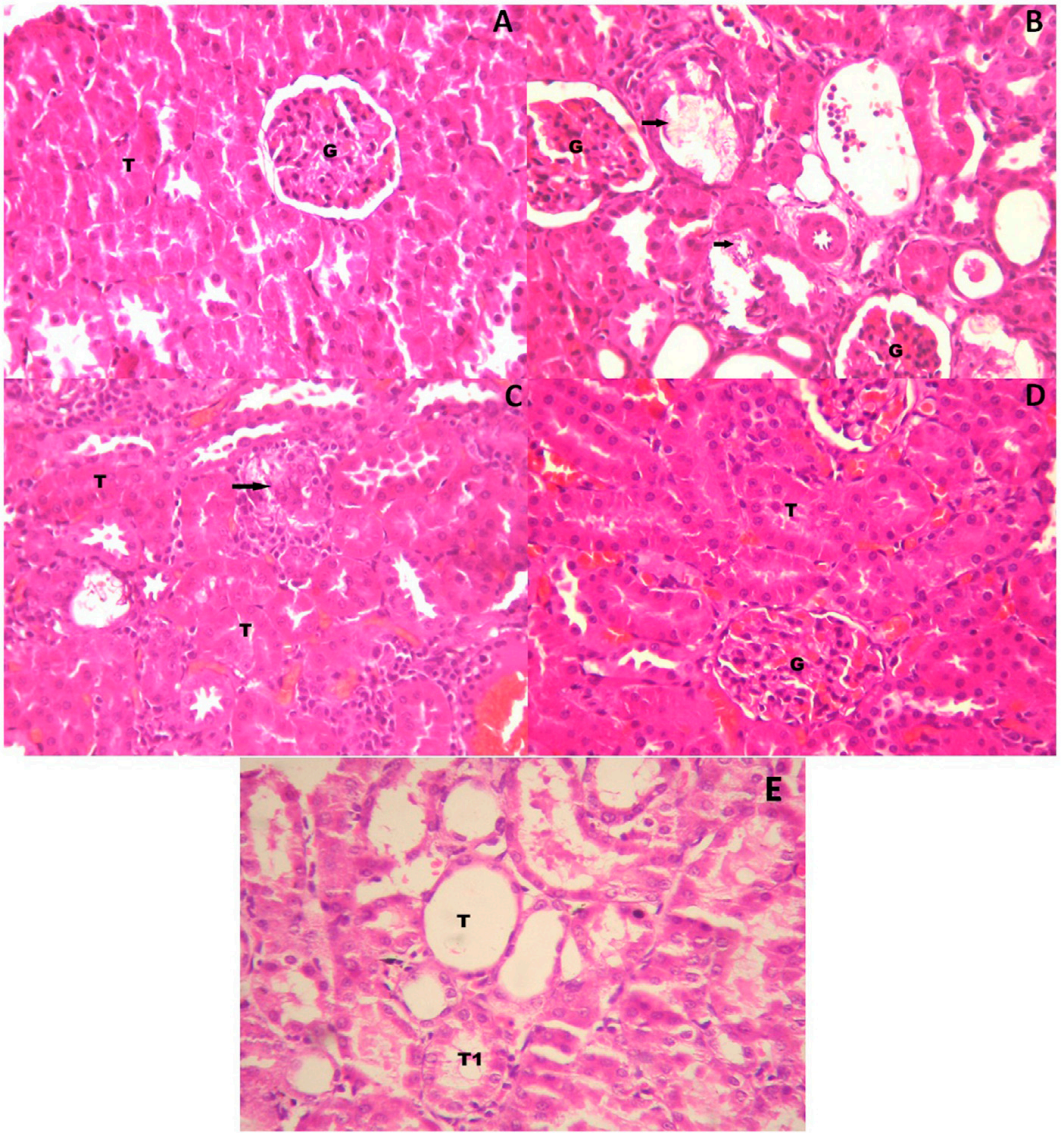
High power photomicrograph of section of kidney **(A)** control group showing a normal glomerulus and tubules **(B)** urolithiatic group showing damaged proximal tubules (arrow) with deposits of refractile crystals and loss of tubular epithelium. The distal tubules (DT) are not affected, glomerulus shows no damage **(C)** urolithiatic group treated with SPP 700 mg/kg/day showing a single damaged proximal tubule (Arrow) with loss of tubular epithelium with several undamaged tubules (T) around it **(D)** Urolithiatic group treated with SPP 1000 mg/kg/day showing a normal glomerulus and tubules. No evidence of tubular damage was seen in this sample. G = Glomerulus, T = Tubule. **(E)** But the Neeri standard group’s rat kidneys showing a few dilated tubules along with a number of undamaged tubules (HE × 40).

Figure 3 shows the histopathological examination of the rat kidneys. The histopathological section of the kidneys of rats in the control group exhibited no abnormalities; glomerulus and proximal tubules were normal (Figure 3A). The kidneys of rats in the toxicant group exhibited damaged proximal tubules, with deposits of refractile crystals and loss of tubular epithelium. The distal tubules were not affected, and glomerulus did not show any damage. It exhibited the characteristic signs of stone development on continuous ingestion of 0.75% EG with 1.0% AC (Figure 3B). The kidneys of rats in the urolithiatic group treated with SPPL (700 mg/kg/day) exhibited a single damaged proximal tubule with loss of tubular epithelium with several undamaged tubules around it (Figure 3C), whereas those treated with SPPH 1000 mg/kg/day exhibited a normal glomerulus and tubules. No evidence of tubular damage was observed in this sample (Figure 3D). However, the kidneys of rats in the neeri standard group exhibited a few dilated tubules along with numerous undamaged tubules (Figure 3E).

### Long Term Oral Toxicity Study of Safoof-e-Pathar Phori 700 mg/Kg

The long-term oral toxicity study of SPP was performed as per the OECD guideline (OECD, 2020) for 90 days. For determining the long-term oral toxicity of the animals, observations were noted based on the following aspects: cage side observation of all animals, mortality record, body weight record, hematological observation, and liver and renal function test with histopathological observations of the heart, kidney, liver, spleen, and stomach.

**FIGURE 4:**
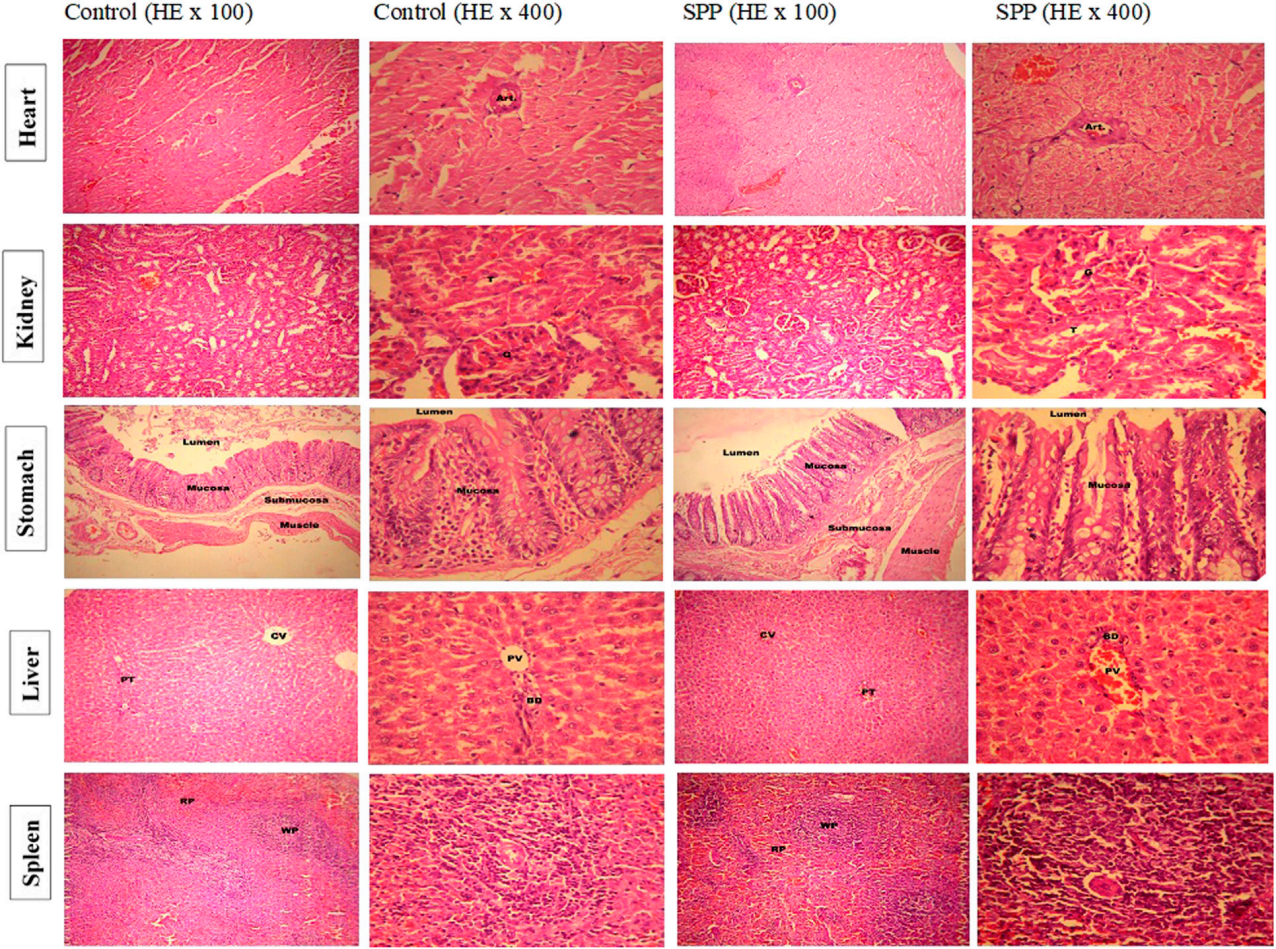
Low and high-power photomicrograph of SPP treated rats showed the normal histological appearance without any significant change in heart, kidney, liver, spleen and stomach compare to normal control rats. Histopathological examination of heart tissue showed normal appearance of cardiac muscle fibers and no evidence of necrosis or myocardial damage is seen. Cardiac muscle fibers with striated cytoplasm and central oval nuclei. A coronary vessel (Art.) cut in cross section is also seen. Histopathological examination of kidney tissues showed normal histopathological appearance of renal parenchyma, glomerular tubule in all treatment groups including control. The histopathological examination of stomach tissues showed normal histological appearance of different layers of stomach walls and mucosa of the stomach showing gastric glands in photomicrographs of all treatment groups including control. Histopathological examination of liver tissues showed normal histological appearance of portal triad (PT), central vein (CV) (100×), bile duct (BD) and portal vein (PV) (400×) in all treatment group including control. The histopathological examination of spleen tissues showed normal histological appearance of white pulp (WP), red pulp (RP) (100×), areas in the splenic parenchyma. Splenic arteriole (SA) and lymphocytes in photomicrographs of all treatment groups including control.

The cage side observation of animals subjected to toxicity was performed based on several parameters on each day up to 90 days, but no abnormality was observed during the complete course of the study. Of the 20 rats, none showed mortality up to 90 days (Table 3). The body weight record of the rats (Table 4) did not exhibit any irregularity in all the treatment groups. Similarly, 13-weeks treatment of the rats with 700 mg/kg SPP did not exhibit any significant difference in hematological parameters (Table 5) as well as in liver and kidney function tests (Table 5). There was insignificant variation in the wet weight of vital organs in the SPP (700 mg/kg/day)-treated group compared with the control group, and these results were observed in both male and female rats (Table 5). Histopathological analysis of the heart, kidney, liver, spleen, and stomach in the SPP-treated rats revealed the normal histological appearance of all the organs without any significant change compared with NC rats (Figure 4). Thus, SPP was found to be safe after its oral administration at 700 mg/kg for up to 3 months (90 days).

**TABLE 3:** Mortality record of animals in control and treatment group.

Days	Control male	Control female	SPP 700 mg/kg male	SPP 700 mg/kg female
**Week 1**	**-**	**-**	**-**	**-**
**Week 2**	**-**	**-**	**-**	**-**
**Week 3**	**-**	**-**	**-**	**-**
**Week 4**	**-**	**-**	**-**	**-**
**Week 5**	**-**	**-**	**-**	**-**
**Week 6**	**-**	**-**	**-**	**-**
**Week 7**	**-**	**-**	**-**	**-**
**Week 8**	**-**	**-**	**-**	**-**
**Week 9**	**-**	**-**	**-**	**-**
**Week 10**	**-**	**-**	**-**	**-**
**Week 11**	**-**	**-**	**-**	**-**
**Week 12**	**-**	**-**	**-**	**-**
**Week 13**	**-**	**-**	**-**	**-**
**Mortality**	**0**/**5**	**0**/**5**	**0**/**5**	**0**/**5**

SPP-Safoof-e-Pathar phori.

**TABLE 4:** Body weight records of animals in male and female rats orally treated with SPP 700 mg/kg and control group.

Dose	Male (g)		Female (g)	
	Control	SPP 700	Control	SPP 700
**0 Week (**18/07/2010)	148 ± 13.26	142 ± 11.66	133.43 ± 38.36	128.22 ± 36.55
**Week 1 (**25/07/2010)	149 ± 14.96	147 ± 11.66	136.16 ± 38.60	131.97 ± 37.66
**Week 2 (**01/08/2010)	158 ± 14.35	157 ± 11.66	142.69 ± 40.84	141.13 ± 40.33
**Week 3 (**8/08/2010)	163 ± 14.35	164 ± 11.13	147.69 ± 42.45	147.92 ± 42.20
**Week 4 (**15/08/2010)	169 ± 13.56	175 ± 8.94	152.29 ± 43.86	157.41 ± 45.49
**Week 5 (**22/08/2010)	173 ± 14.35	183 ± 6.63	156.02 ± 44.83	163.05 ± 47.41
**Week 6 (**29/08/2010)	180 ± 12.64	189 ± 6.63	161.88 ± 47.14	169.63 ± 49.74
**Week 7 (**5/09/2010)	184 ± 12	195 ± 7.07	166.33 ± 48.60	175.58 ± 51.33
**Week 8 (**12/09/2010)	189 ± 10.19	201 ± 8	171.59 ± 50.05	182.41 ± 53.03
**Week 9 (**19/09/2010)	196 ± 11.57	209 ± 8	177.71 ± 51.42	189.75 ± 55.29
**Week 10 (**26/09/2010)	202 ± 12.08	214 ± 3.74	183.67 ± 53.28	193.97 ± 57.71
**Week 11 (**3/10/2010)	208 ± 12.88	221 ± 3.74	190.07 ± 54.88	199.97 ± 59.50
**Week 12 (**10/10/2010)	214 ± 13.92	228 ± 4	196.49 ± 56.62	206 ± 61.18
**Week 13 (**17/10/2010)	225 ± 12.24	236 ± 3.74	206.85 ± 59.57	214.14 ± 63.64

**TABLE 5:** Results of hematological, liver, renal and organ weight assessment of control and SPP 700 mg/kg group.

Test	Male		Female	
	Control	SPP 700	Control	SPP 700
**Erythrocyte count (mill/C.mm)**	5.81 ± 0.01	7.30 ± 0.06	5.31 ± 1.59	6.61 ± 1.97
**Hemoglobin (g/dl)**	9.84 ± 0.16	13.22 ± 0.08	9.52 ± 2.88	11.70 ± 3.53
**Total leukocytes**	11.84 ± 0.10	11.18 ± 0.06	10.49 ± 3.16	10.14 ± 3.04
**Bilirubin (mg/dl)**	00.93 ± 0.02	0.88 ± 0.01	00.85 ± 0.25	00.79 ± 0.23
**SGPT (U/L)**	30.92 ± 0.66	69.68 ± 0.93	27.69 ± 8.21	63.55 ± 18.89
**SGOT (U/L)**	35.26 ± 0.34	67.4 ± 0.61	32.24 ± 9.63	60.95 ± 18.22
**ALP (U/L)**	75.11 ± 0.28	25.34 ± 0.53	67.90 ± 20.42	14.73 ± 10.67
**Total Protein(g/dL)**	00.13 ± 0.01	04.70 ± 0.08	05.71 ± 1.69	04.26 ± 1.26
**Albumin (g/dl)**	03.12 ± 0.14	1.30 ± 0.02	02.77 ± 0.80	01.18 ± 0.35
**Blood Urea (mg/dl)**	34.6 ± 1.01	49.22 ± 0.16	31.30 ± 9.19	81.90 ± 124.3
**Creatinin (mg%)**	00.73 ± 0.02	00.44 ± 0.01	00.66 ± 0.19	00.40 ± 0.11
**Serum Uric acid(mg/100 ml)**	07.06 ± 0.10	08.02 ± 0.11	06.40 ± 1.90	07.27 ± 2.16
**Sodium (meq/L)**	143.8 ± 2.31	140 ± 1.41	130.42 ± 38.70	127.03 ± 37.95
**Potassium (meq/L)**	04.16 ± 0.13	07.24 ± 0.13	03.69 ± 1.09	06.48 ± 1.93
**Kidney**	0.68 ± 0.01	0.69 ± 0.007	0.61 ± 0.18	0.63 ± 0.18
**Spleen**	0.31 ± 0.01	0.31 ± 0.01	0.28 ± 0.08	0.29 ± 0.08
**Heart**	0.40 ± 0.01	0.41 ± 0.006	0.37 ± 0.11	0.37 ± 0.11
**Liver**	2.01 ± 0.08	1.94 ± 0.10	1.87 ± 0.54	1.80 ± 0.51
**Stomach**	0.38 ± 0.02	0.37 ± 0.007	0.35 ± 0.10	0.34 ± 0.10

## Discussion

The most commonly used medical therapies in the management of urolithasis include calcium channel blockers, steroids, and α_1_ adrenergic blockers (Shekar and Patki, 2011), which exhibit some adverse effects. Some herbal remedies have been used in the management of urinary calculi; however, their efficacy has not been scientifically proven. With the understanding of various pathophysiological functions underlying renal stones and the mechanism of herbal remedies that can play a role in preventing the formation and management of urinary calculi, phytotherapy might be a better alternative in the preventive and curative management of urinary calculi. Oral citrate is the most commonly used medicinal agent in the prevention of urinary calculi (Gurocak et al., 2006). Because of the adverse effects of these agents, alternative management modalities comprising herbal remedies have been significant agents in the treatment (Gurocak et al., 2006). The use of medicinal plants to treat diseases is as old as civilization itself. Plants provide crude drugs, which are used without any modification, and numerous chemical constituents can be used for the synthesis of new drugs with better pharmacological effects (Potterat and Hosttettman, 1995; Zaidi and Ahmad, 2016). Additionally, the World Health Organization (WHO) has highlighted the development and application of herbal and traditional medicines considering their beneficial effects, such as cost-effectiveness and the avoidance of side effects of synthetic medicines. The WHO has estimated that approximately 80% of the population living in the developing countries rely on traditional medicines for their healthcare needs (WHO, 2002).

The present study was performed to evaluate the antiurolithiatic effects of SPP on EG-induced urolithiasis in male Wistar rats used by several investigators. (Bashir and Gilani, 2009; Pareta et al., 2011; Aggarwal et al., 2012; Lin et al., 2012; Nanu et al., 2012; Saeidi et al., 2012; Khan et al., 2016).

EG is a metabolic precursor of oxalate. EG is oxidized to glycolic acid and further to oxalic acid. Administration of EG causes hypercalciuria, leading to urolithiasis. Administration of EG causes hypercalciuria and hyperoxaluria to induce urolithiasis (Verma et al., 2009). AC ingestion, which induces metabolic acidosis, has been used in conjunction with EG to promote the deposition of CaOx crystals in the rat kidneys. Various doses of AC in combination with EG resulted in CaOx depositions in the rat kidneys within 7 days (Khan et al., 2016). After 21 days, crystalluria analysis of rats in the EG-induced urolithiasis group revealed that untreated rats exhibited bigger crystals than treated rats (Bashir and Gilani, 2009). Studies have consistently reported that calculi induced by hyperoxaluria causes an increase in oxalate and a decrease in calcium excretions in the toxicant group (Fan et al., 1999; Park et al., 2007); SPP treatment produced reversible effects in a dose-dependent manner.

Results of the urinary calcium level showed a significant (*p* < 0.001 vs control) elevation in the toxicant group, whereas sodium and potassium level significantly (*p* < 0.001 vs control) reduced compared to the control group. Sodium and potassium levels significantly (*p* < 0.001 vs toxicant) increased, and calcium levels were decreased in the urine of the rats treated with SPP compared with the toxicant group. Comparison of the SPP and Neeri group showed an insignificant difference. This suggests an increased urinary loss of electrolytes (Hess and Kok, 1996). In urolithiasis, creatinine and BUN accumulate in blood. In this study, we observed that serum creatinine and BUN levels were significantly (*p* < 0.001 vs control) elevated in the serum of rats with EG- and AC-induced urolithiasis (toxicant group). It is suggested that EG and AC cause renal tubular damage and lower glomerular filtration rate. SPP-treated rats significantly (*p* < 0.001 vs. toxicant) reduced the BUN and serum creatinine levels compared with the control group at the dose of 700 and 1,000 mg/kg, and these results were similar to those of the standard drug Neeri.

The toxicant group showed a significantly (*p* < 0.001 vs control) elevated level of TBARS compared to the control group. Lipid peroxidation was measured as a Nano-gram of TBARS per mg of protein level in kidney tissue. Lipid peroxidation is another critical cause of injury that occurs during urolithiasis. Increased TBARS levels due to increased oxidative stress, decreased antioxidant enzymes and GSH levels in kidneys, and impaired kidney functions. Oxalate has been reported as the major stone forming component causing peroxidative injury to the renal epithelial cells (Kato et al., 2007). The SPP treatment protected against various injuries associated with oxidative stress. The biochemical alterations were supported by histopathological interpretations of the kidney (Muthukumar and Selvam, 1998). The urolithiasis toxicant group exhibited the presence of refractile crystals in the tubular epithelial cell and damage to the proximal tubular epithelium, whereas groups treated with SPP along with EG and AC exhibited protection, as evident from the reduction in tubular cell damage and normal kidney histology. Our results suggest that SPP has both protective and preventive effects in rats with EG- and AC-induced urolithiasis, which is consistent with previous studies (Saha and Verma, 2011). Zaidi and Ahmad (2016) reported that SPP is an effective and safe non-invasive polyherbal remedy for patients with urolithiatic, with no adverse reactions.

Medicinal plants and herbal medicines are progressively required by patients as a source of prescription drugs, in the form of active constituents, in developed and developing countries. They have been shown to possess undeniable and tangible therapeutic benefits with limited toxicity because of their long-term use as traditional medicines. The WHO claims that safety is a critical parameter of herbal medicines in the quality control of healthcare products (Tatke et al., 2012). Toxicity studies are considered to be important during new drug development, keeping in mind that herbal medicines are frequently used indiscriminately without considering their potential side effects, which can vary from mild–severe to life-threatening (World Health Organization, 1987; World Health Organization, 2000). Various herbal preparations have been shown to be beneficial in treating kidney disease; however, the toxicity and safety data for many of these herbal treatments are not available (Tatke et al., 2012).

In this study, the long-term oral toxicity of SPP was evaluated in albino Wistar rats using biochemical, hematological, and histopathological parameters. Weight gain and behavioral activity was similar to the control animals in all single-dosed males and females during the study period. There were no marked abnormal changes observed throughout the study period. Hematology in rats at a single dose of SPP exhibited insignificant differences compared to the control group. Additionally, the functioning of major organs (heart, kidney, liver, spleen, and stomach) was found to be similar to that in the control group. Histopathological examination of the heart, kidney, liver, spleen, and stomach did not exhibit any changes. Oral SPP ingested at 700 mg/kg for up to 3 months resulted in normal growth with no changes in hematological, hepatic, or renal functioning parameters. There was no evidence of abnormal histology. Thus, long-term daily oral consumption of SPP was found to be safe in Wistar rats, and it may be safe for clinical use as an antiurolithiatic formulation.

## Conclusion

The results indicated that the administration of the traditional Unani poly-herbomineral formulation SPP reduced and prevented the growth of urinary stones in rats with EG- and AC-induced urolithiasis. The toxicity study indicated no serious signs and significant changes in the physical, hematological, biochemical, and histopathological parameters after 90-days administration of SPP (700 mg/kg). SPP was found to be safe for oral administration at 700 mg/kg for up to 3 months. Hence, SPP was observed to be a curative and safe poly-herbomineral formulation, useful in the prevention and management of urolithiasis. Further studies at lower dose levels and using extracts of the formulation to reduce the dose are necessary to make it more pharmacologically relevant and to elucidate the mechanism underlying the pharmacological effect of SPP.

## Data Availability

The original contributions presented in the study are included in the article, further inquiries can be directed to the corresponding author.

## References

[B1] Aggarwal A Singla SK Gandhi M Tandon C (2012). Preventive and curative effects of *Achyranthes aspera* Linn. extract in experimentally induced nephrolithiasis. Indian J. Exp. Biol. 50, 201–8. 22439435

[B2] Ahmad W. Ali A. Ali M. Mir S. R. Zaidi S. M. A. Ahmad S. (2017). New fatty acid and aromatic monoterpenic esters from the leaves of *Didymocarpus pedicellata* R. Br. Indian Drugs. 54 (11), 28–32.

[B3] Ahmad W. Khan W. Khan M. S Mujeeb M. Zaidi S. M. A. Ahmad S. (2016). Quality control analysis of Safoof-e-Pathar phori: antiurolithiatic formulation. Drug Dev. Ther. 7 (1), 20–25. 10.4103/2394-6555.180163

[B4] Ahmad W. Mujeeb M. Zaidi S. M. A. Ahmad S. (2013). Current strategy for research on quality identification of *Rheum emodi* Wall. rhizome. Int. J. Drug Dev. Res. 5, 1–10.

[B5] Ahmad W. Mujeeb M. Zaidi S. M. A. (2010). Quality control analysis of seed of *Dolichous biflorus* Linn. Int. J. Drug Dev. Res. 2, 669–674.

[B6] Ahmad W. Parveen R. Mujeeb M. Zaidi S. M. A. (2020). Comparative fingerprint profiling of Unani polyherbomineral (Safoof-e-Pathar phori) formulation by HPTLC, HPLC, and GC-MS. J. AOAC Int. 103, 659–668. 10.5740/jaoacint.19-0286 31619315

[B7] Ahmad W. T. Tamboli E. Ali A. Amir M. Zaidi S. M. A. Ahmad S. (2019). *Didymocarpous pedicellatus* R. Br.: qualitative and quantitative GCMS approach for quality control in traditional poly-herbal formulation with in vitro antioxidant and antimicrobial activity. Orient. J. Chem 35, 648–657. 10.13005/ojc/350220

[B8] Ahmad W. Zaidi S. M. A. Ahmad S. (2014). Quality control analysis of *Didymocarpous pedicellata* R. Br. Indian J. Traditional Knowledge. 13, 175–180.

[B9] Ahmad W. Zaidi S. M. A. Mujeeb M. Ansari S. H. Ahmad S. (2014). HPLC and HPTLC methods by design for quantitative characterization and *in vitro* anti-oxidant activity of polyherbal formulation containing *Rheum emodi* . Journal of Chromatographic Science 52, 911–918. 10.1093/chromsci/bmt123 23978770

[B10] Ahmad W. Zaidi S. M. A. Ahmad S. (2015). Validated UPLC-Q-TOF-MS method for quantitative determination of emodin in rhizome of *Rheum emodi* Wall. ex Meissn. and its traditional polyherbal formulation using three different extraction techniques. Ann. Phytomedicine 4, 68–73.

[B11] Anonymous (2006). The wealth of India second supplement series raw material. 58.

[B12] Anonymous (2007a). The Unani Pharmacopoeia of India. 58–59.

[B13] Anonymous (2007b). The Unani Pharmacopoeia of India. 91–92.

[B14] Anonymous (1986). Qarabadeen majeedi all India Unani tibbi conference. Delhi.

[B15] Asplin J. R. Favus M. J. Coe F. L. (1996). “Nephrolithiasis,” in Brenner and Rector’s the kidney. Editor Brenner B. M. 5th ed. (Philadelphia: Saunders). 1893–1935.

[B16] Bashir S. Gilani A. H. (2009). Antiurolithic effect of Bergenia ligulata rhizome: an explanation of the underlying mechanisms. Journal of Ethnopharmacology 122, 106–116. 10.1016/j.jep.2008.12.004 19118615

[B17] Chandhoke P. S. (2002). When is Medical Prophylaxis Cost-effective for Recurrent Calcium Stones?. The Journal of Urology 168, 937–940. 10.1097/00005392-200209000-00009 12187194

[B18] Dubey N. Mehta R. S. Saluja A. K. Jain D. K. (2008). Quality assessment of Khusta-e-Gaodanti: a traditional Unani medicine. Asian J. Res. Chem. 1, 46–50.

[B19] Fan J. Glass M. A. Chandhoke P. S. (1999). Impact of ammonium chloride administration on rat ethylene glycol urolithiasis model. Scanning Microsc. 13, 299–306.

[B20] Gürocak S. Küpeli B. (2006). Consumption of historical and current phytotherapeutic agents for urolithiasis: a critical review, Journal of Urology 176, 450–455. 10.1016/j.juro.2006.03.034 16813863

[B21] Hardik G. Maunik C. Pinakin J. (2016). Diuretic and antiurolithiatic activities of an ethanolic extract of Acorus calamus L. rhizome in experimental animal models. J. Tradit Complement. Med. 6(4), 431–436. 10.1016/j.jtcme.2015.12.004 27774431 PMC5067935

[B22] Hess B. Kok D. J. (1996). Nucleation growth and aggregation of crystals. In: Kidney stones, medical and surgical management. Editors: Coe F. L. Favus M. J. Pak C. Y. Parks J. H. Philadelphia, PA, USA: Lippincott-Raven. 3–32.

[B23] Ikshit S. Washim K. Rabea P. Md J. A. Iftekhar A. Mohd H. R. A. (2017). Antiurolithiasis activity of bioactivity guided fraction of bergenia ligulata against ethylene glycol induced renal calculi in rat. Biomed. Res. Int. 1969525. 10.1155/2017/1969525 28349055 PMC5352974

[B24] Kato J. Ruram A. A. Singh S. S. Devi S. B. Devi T. I. Singh W. G. (2007). Lipid peroxidation and antioxidant vitamins in urolithasis. Indian J. Clin. Biochem. 22, 128–130. 10.1007/bf02912895 23105666 PMC3454268

[B25] Kaur T. Bijarnia R. K. Singla S. K. Tandon C. (2009). *In vivo* efficacy of *Trachyspermum ammi* anticalcifying protein in urolithiatic rat model. Journal of Ethnopharmacology 126, 459–462. 10.1016/j.jep.2009.09.015 19781619

[B26] Khaling M. Suresh K. Vandana R. (2014). Current scenario of urolithiasis and the use of medicinal plants as antiurolithiatic agents in Manipur (North East India): a review. Int. J. Herb. Med. 2, 1–12.

[B27] Khan M. A. Kumar S. Gupta A. Ahmad S. (2016). Screening of two new herbal formulations in rodent model of urolithiasis. Drug Dev. Ther. 7, 34–38. 10.4103/2394-6555.180160

[B28] Kumar S. Pandey A. K. (2014). Medicinal attributes of Solanum xanthocarpum fruit consumed by several tribal communities as foodn *in vitro* antioxidant, anticancer and anti HIV perspective. BMC Complement. Altern. Med. 14, 112. 10.1186/1472-6882-14-112 24678980 PMC3973604

[B29] Lin W.-C. Lai M.-T. Chen H.-Y. Ho C.-Y. Man K.-M. Shen J.-L. (2012). Protective effect of *Flos carthami* extract against ethylene glycol-induced urolithiasis in rats. Urol. Res. 40, 655–661. 10.1007/s00240-012-0472-4 22398437

[B30] Mandavia DR Patel MK Patel JC Anovadiya AP Baxi SN Tripathi CR (2013). Anti-urolithiatic effect of ethanolic extract of Pedalium murex linn. fruits on ethylene glycol-induced renal calculi. Urol. J. 10, 946–52. 24078501

[B31] Michell R. Med J. R. Soc. (1989). Urolithiasis-historical, comparative and pathophysiological aspects: a review. J. Mol. Sci. 82, 669–672. 10.1177/014107688908201112 PMC1292373 2687468

[B32] Mina C. N. Marziyeh H. Roja R. Mohammad H. F. Stéphane Z. Seyed M. N. (2018). Dietary plants for the prevention and management of kidney stones: preclinical and clinical evidence and molecular mechanisms. Int. J. Mol. Sci. 19(3) 765. 10.3390/ijms19030765 PMC5877626 29518971

[B33] Moe O. W. (2006). Kidney stones: pathophysiology and medical management. The Lancet 367, 333–344. 10.1016/s0140-6736(06)68071-9 16443041

[B34] Muthukumar A. Selvam R. (1998). Role of glutathione on renal mitochondrial status in hyperoxaluria. Mol. Cel Biochem 185, 77–84. 10.1023/a:1006817319876 9746214

[B35] Nanu R. R. Dipak B. Havagiray R. C. Sanjeev R. Muchandi I. S. Ramesh C. (2012). Anti-urolithiatic effects of *Punica granatum* in male rats. J. Ethnopharmacol. 140, 234–238. 10.1016/j.jep.2012.01.003 22285521

[B36] OECD (2020). OECD guidelines for the testing of chemical. Repeated dose 90 days oral toxicity study in rodents. Available at: https://www.oecd-ilibrary.org/docserver/9789264070707-n.pdf?expires=1616865109&id=id&accname=guest&checksum=2EECD0312717F23C7B804C6EA616492B (Accessed July 15, 2020).

[B37] Pareta SK Patra KC Mazumder PM Sasmal D (2011). Aqueous extract of *Boerhaavia diffusa* root ameliorates ethylene glycol-induced hyperoxaluric oxidative stress and renal injury in rat kidney. Pharm. Biol. 49, 1224. 10.3109/13880209.2011.581671 [Epub ahead of print] 21846174

[B38] Park HK Jeong BC Sung MK Park MY Choi EY Kim BS (2007). Reduction of oxidative stress in cultured renal tubular cells and preventive effects on renal stone formation by the bioflavonoid quercetin. J. Urol. 179, 1620–6. 10.1016/j.juro.2007.11.039 18295251

[B39] Park S. Pearle M. S. (2007). Pathophysiology and management of calcium stones. Urologic Clinics of North America 34, 323–334. 10.1016/j.ucl.2007.04.009 17678983

[B40] Patankar S. Dobhada S. Bhansali M. Khaladkar S. Modi J. (2008). A prospective, randomized, controlled study to evaluate the efficacy and tolerability of Ayurvedic formulation “varuna and banana stem” in the management of urinary stones. J. Altern. Complement. Med. 14, 1287–1290. 10.1089/acm.2008.0189 19040391

[B41] Potterat O. Hosttettman K. (1995). “Plant source of natural drugs and compounds”. Encyclopedia of environmental biology. Editor Nierenberg W. A. (London: Academic Press), 13, 139–153.

[B43] Saeidi J Bozorgi H Zendehdel A Mehrzad J (2012). Therapeutic effects of aqueous extracts of *Petroselinum sativum* on ethylene glycol-induced kidney calculi in rats. Urol J 9, 361–6. 22395833

[B44] Saha S Verma RJ (2011). *Bergenia ciliata* extract prevents ethylene glycol induced histopathological changes in the kidney. Acta Pol. Pharm. 68, 711–5. 21928716

[B45] Sathish R. Natarajan K. Nikhad M. M. (2010). Effect of *Hygrophila spinosa* tanders on ethylene glycol induced urolithiasis in rats. Asian J. Pharma. Clin. Res. 3, 61–63. 10.4103/0253-7613.100402

[B46] Shekar K. M. G. Patki P. S. (2011). Evaluation of an Ayurvedic formulation (Cystone), in urolithiasis: a double blind, placebo-controlled study. Eur. J. Integr. Med. 3, 23–28. 10.1016/j.eujim.2011.02.003

[B47] Srinivas S. Venkanna B. Mohan E. M. Mohan C. K. (2012). Urolithiasis: overview. Int. J. Pharma RBA. 1, 20–31.

[B48] Srinivasa A. K. B. Kuruba L. Saran G. S. (2013). Antiurolithiatic activity of Gokhsuradichuran, an Ayurvedic formulation by *in-vitro* method. Adv. Pharm. Bull. 3, 477–479. 10.5681/apb.2013.080 24312883 PMC3848215

[B49] Soundararajan P. Kahesh R. Ramesh T. Hazeena V. (2006). Effect of *Aervalanta* on calcium oxalate urolithiasis in rats. Indian J. Exp. Biol. 44, 981–986. 10.2514/1.17320 17176671

[B50] Sutherland JW Parks JH Coe FL (1985). Recurrence after a single renal stone in a community practice. Miner Electrolyte Metab. 11, 267–9. 4033604

[B51] Tatke P. Deshpande S. Nidhiya I. S. R. (2012). Safety profile of a polyherbal formulation (gynocare capsules) in female rats by subchronic oral toxicity study. Toxicol. Int. 19, 106–1110. 10.4103/0971-6580.97196 22778505 PMC3388751

[B52] Verma N. K. Patel S. S. Saleem T. S. M. Christina A. J. M. Chidambaranathan N. (2009). Modulatory effect of NONI-Herbal formulation against ethylene glycol-induced nephrolithiasis in albino rats. J. Pharm. Sci. Res. 1, 83–89.

[B53] World Health Organization (2000). General guidelines for methodologies on research and evaluation of traditional medicine. Geneva: World Health Organization.

[B54] World Health Organization (1987). Principles for the safety assessment of food additives and contaminants in food. In IPCS environmental Health criteria 70. Geneva: World Health Organization.

[B55] World Health Organization (2002). World Health organization monographs on selected medicinal plants. Geneva: World Health Organization. 159. 10.1111/j.2042-7166.2000.tb02454.x

[B56] Zaidi S. M. A. Ahmad W. (2016). Randomized single-blind clinical evaluation of Safoof-e-Pathar phori in urolithiasis patients. Drug Dev. Ther. 7 (2), 92–95. 10.4103/2394-6555.191151

